# Dr. Cawrwardine, on the Action of Muscles

**Published:** 1808-04

**Authors:** H. H. Carwardine

**Affiliations:** Surgeon. Thaxted, Essex


### Medical and Physical Journal.

VOL. XIX.] APRIL, 1808. [NO. 110.

*Printed for R. PHILLIPS, by W. Thorne, Red Lion Court, Fleet Street, London*

### To Dr. BATTY.

DEAR SIR,

I Send you the following observations, not because they are new or original, but because I conceive them to be useful. The principles which I have laid down and endeavoured to illustrate, are now taught, I believe, by most of the professors in the schools; whether they are sufficiently dwelt upon and enforced by them, I dare not take upon me to determine; but the more I converse with my professional brethren, and especially the more I *see* of their practice, the more I feel convinced that these principles are not *generally* known, and still less generally are they *adverted to* in *practice*: and, although they may be taught in the schools, yet I know of no *writer* who has treated this subject with such force and interest, as particularly to arrest the attention of his reader, and make him feel the soundness and importance of the doctrine.

I am, Dear Sir,

Your obliged Friend,

*Thaxted, Essex.* H. H. CARWARDINE, Surgeon.

---

### *Some Observations on the Action of Muscles, as preventive of the Reduction of Luxations; and on the most simple Method of overcoming that Action.*

I HAVE been much surprised among the various authors whom I have consulted, and even the professional men with whom I have conversed upon the subject, to find the genuine principles on which the reduction of luxations should be conducted, so little adverted to in practice. I was particularly led to make this remark by the perusal of Mr. Hey's observations on the subject; for although he seems to come nearer the true principles than any author I

have seen, (vide p. 298,) yet his practice in the cases which he afterwards relates, appears to be very little guided by them. What I mean by these principles, is simply this,—a correct knowledge of those causes which prevent our putting a bone, when out, into its place again, and the most simple and obvious mode of overcoming this difficulty.

Surgical writers abound in descriptions of the various luxations to which each bone is liable; the situation into which the head of it may be thrown; and the probable injury sustained by the capsular ligament, and other parts which enter into the composition of a joint; and they are also very ample in describing the direction in which the force is to be applied for the reduction of each of these varieties. A correct knowledge of the anatomy of the joints, and of the relative situation of parts in their natural position, will always supply us with every necessary information on this head, and it is not therefore my intention to dwell upon it.

To come then at once to the object of this paper, and to illustrate by example, I shall suppose a case. A man has luxated his shoulder; the head of the bone lies in the axilla: what prevents my putting it into the socket again? It appears that I have nothing to do but to pull forward the head of the bone, till it comes on a line with the cavity which is to receive it, then make a lever of it by putting my finger just beneath the neck of the bone for a fulcrum, depressing the elbow with the other hand, and thus elevate the head at once into its place!* Now, the first difficulty which I find oppose itself to this very obvious practice, is, that the scapula being a sort of loose floating bone, the same force which draws down the head of the humerus, draws down also the cavity which is to receive it, so that the ball and socket still maintain the same relative situation as before my endeavour; the remedy for this is obvious, the scapula must be fixed to the trunk, and the trunk also will need to be fixed, in order to give the necessary

* And were it not for the action of the muscles, it would doubtless be just as simple a matter as I have here represeuted it; for in those fortunate cases where the bone is suddenly reduced by a sort of coup de main, this is precisely what happens; the attention of the patient is amused; the muscles are off their guard; their action is eluded, and their watchful irritability deceived by a little gentle handling and motion of the limb; when by a sudden and dexterous performance of the combined motions I have detailed above, the luxation is reduced, because the muscles have not opposed it.

cessary resistance to the extension of the arm. The scapula being fixed then, we now find a new obstacle to our endeavours; for the muscles hold the head of the bone fast in its situation, and it cannot be moved till this power is overcome. Let us advert for one moment to the nature of this power, the most extraordinary in the animal machine. The muscular power we find in its greatest and most violent action, sufficient to snap the tendons, and break the very bones; and it invariably acts with an exertion proportionate to the resistance it has to overcome, as far as the limits of its action extend; but let this exertion be greater or less, it can only be sustained for a given time, when the muscular power becomes exhausted, and relaxation takes place. The most familiar instance of this, is the holding a small weight at arm's length for a given time; in less than five minutes the deltoid muscle becomes tired and painful, its power exhausted, and the arm drops. If this trifling weight, long continued, can exhaust the muscular power, what need of that immense force, and distressing violence, which we daily see employed in the reduction of dislocated joints? Three or four people are set to pull at a towel fastened round the limb; they exert themselves under the idea that the muscles must yield to their united efforts; but thus, great pain is excited, which we know to be a vast stimulus to muscular action, (particularly to that spasmodic action, which it should be our chief object to evade); the muscles do not yield, the arms of the assistants themselves are tired, they relax the extension; in this moment the muscles gain fresh vigor; again extension is made, and again the muscular power effectually resists it, till in this way the patient, surgeon, and assistants are all worried and fatigued; the patient begs a respite, and the surgeon almost ashamed to proceed, directs bleeding, the warm bath, and even a poultice to the joint, that he may lower a power which he imagines he cannot otherwise overcome. All this I have seen practised, and by those too who hold a high rank in their profession! But surely this is not consistent with the genuine principles of our art; these rude attempts at overcoming the muscular power, (if I may be allowed the expression,) by *main force* of *surgery,* when the regular and unremitting extension of a force equal to a very few pounds weight would infallibly exhaust it. I shall illustrate what I have here advanced with a single case, which shall conclude the subject.

John Bridge, a stout muscular man, about 40 years of

 age, age, by trade a carter, dislocated his shoulder by the sudden tossing of the head of one of his horses, as he was adjusting a part of the harness; the accident happened in the country; and the surgeon, after some ineffectual attempts to reduce it, bound it up and gave him an embrocation, without explaining to him very clearly what had happened. Not finding his arm get better, and having occasion to come to Smithfield with his cart, he called at ——— Hospital about a *fortnight* after the accident had happened. One of the senior surgeons was going round and saw him, explained to him the nature of the accident, and proceeded to the reduction of the joint. After repeated and violent attempts, which proved ineffectual, it was proposed to the man to remain in the Hospital till the following day, and in the interim to lose some blood, take a cathartic, and keep a relaxing poultice to the joint. Soon after the surgeon had quitted him, I happened to come into the ward, when the man was pointed out to me by Mr. W——, (a brother dresser,) who informed me of what I have above related. The man was about to leave the hospital, as his business would not admit of his following the advice of the surgeon.

I had had some previous conversation with Mr. W—— on the *principles* on which the reduction of luxations should be conducted; and as the man was about to quit the hospital unrelieved, we agreed, that if we could prevail upon him, he would be a very fair subject for an experiment. Having explained myself as well as I could to the comprehension of our patient, he agreed to our proposal, and accompanied us to my lodgings, where having provided the necessary apparatus, the man was seated on a stool, the scapula and trunk firmly fixed, and a set of pullies accurately adjusted and secured to the condyles of the humerus for the purpose of making extension. (The head of the bone was beneath the pectoral muscle.) The cord was now tightened with a force just sufficient to counteract the gravity of the limb, and prevent its falling to his side; Mr. W—— with one hand held the wrist to preserve the fore-arm bent, and with the other held the cord to keep it from relaxing, but without tightening it; I took charge of the joint. This extension, which I think could not exceed the force of 16 or 18 pounds weight, was continued for ten minutes without producing any manifest effect; the man experienced little pain, but smiled at our endeavours, and complained of its teazing him to so little purpose. At the end of the ten minutes, as the extension gave so little pain, pain, Mr. W—— slightly increased it; in five minutes more the man grew very restless, would not allow that he suffered pain, but said he felt very faint, begged that we would desist, and asked for water, which I refused, telling him I wished for nothing more than his fainting, by which the muscles would be completely relaxed. At this moment he changed countenance; I could perceive the deltoid muscle tremulous; I could hear something like adhesions giving way; Mr. W—— pulled the cord a little and the arm followed it; I made the necessary motion of the humerus; the cord was suddenly relaxed, and we found the head of the bone in its place, but no sound was produced by it, as is usual. We bound the man's arm to his side, put the fore-arm in a sling, and directed him not to use it for a week. I have never seen or heard of him since.

This Case appears to me so happily illustrative of the particular point which I wished to maintain and enforce, that I shall decline amplifying the subject by any additional instances from my own observation, though I could easily supply them: but there is one Case in Mr. Hey's "Observations in Surgery," which might be regarded almost as a series of experiments instituted for the purpose of proving the utility of the practice above recommended.

A stout muscular fellow, by trade a stone-mason, dislocated his shoulder by a fall from his horse. The reduction was repeatedly attempted by Mr. Hey and his colleagues of the Leed's Infirmary. The man was raised from the ground several times by vertical pullies. Extension was made by several people in different directions. He was laid upon the ground, and one gentleman put his heel in the armpit, and another his foot on the acromion scapulæ. He was twice bled and went into the warm bath, and then had all these operations repeated; when "Mr. Jones suggested the idea of letting his arm fall down without any farther extension. This was done in a gentle manner, but so that the arm fell by its own weight. In this motion the head of the bone slipped into its socket!"

Mr. Pott has given us a few pages on the subject of Luxations. He does not *limit* the force to be applied; but speaks of the propriety of increasing the extension *gradually*. He certainly does not appear aware of the *small* degree of force which will effect the purpose with absolute certainty, if *long continued*.

Boyer, in his celebrated Lectures on Diseases, &c. of the Bones, wherein he speaks at great length on the treatment of Luxations; although he alludes to the principles which I have endeavoured to illustrate and enforce, yet lays very little stress on its advantage, and has no idea of pullies being employed as a means of effecting the reduction with *less force* than by manual exertion; and, therefore, very wisely forbids the use of them altogether. My idea of the use of the pulley, is, that by its assistance we can employ *a force more under our power of regulation; much less in degree, more equal,* and *longer continued,* than by any other means; and that these are the *essential* points to be attended to in overcoming the muscular resistance to the reduction of all luxations.

"It is impossible," (says Boyer) "to assign the precise degree of force to be employed: it is to be varied and proportioned according to the strength of the patient and the number and force of the muscles surrounding the articulation. It has been said, that in reducing a luxation there is occasion for more address than force: it would be true to say that the union of both is necessary. Often *six* assistants accomplish that which three cannot do, and nine or ten accomplish what cannot be done by six. But when the reduction cannot be effected by the number of assistants which we in reason suppose capable of overcoming the resistance, all further attempts must be suspended. The action of pullies, or any other machine analogous to them, would sooner tear the integuments than produce an elongation of the muscles."——

"If these means prove insufficient, the patient is to be repeatedly bled, after short intervals; he is to use the warm bath, and be confined to a very low diet. At the end of twenty-four hours, when he is brought down by this treatment, the luxation, before irreducible in appearance, may now be reduced with facility."

On the principle of exhausting the muscular action, Mr. Boyer writes thus:

"Lastly, one more resource remains, which has sometimes succeeded; it was first employed by Lecat, in a luxation of the jaw, and consists in fatiguing by continual action, the muscles which surround the luxated bone. It is well known that the contractile faculty of our organs is exhausted by exercising them too long, and that the frequent repetition of their contractions necessarily brings about a collapse: the surgeon just cited availed himself of this fact. The levatores muscles of the lower jaw were spasmodically contracted in a case of luxation of that bone, and would not admit of having it brought down. Lecat introduced a small stick between the teeth, and making making use of it as a lever, combated the action of the muscles, until they fell into a state of atony, and allowed him to accomplish the reduction. David has derived similar advantages from the same practice, in luxations of the thigh and arm. This circumstance enables us to explain why a luxation that has resisted the fruitless efforts of an intelligent surgeon, aided by a sufficient number of assistants, afterwards yields to a much less considerable degree of force, used by a less dexterous practitioner."

I have ventured to encroach upon the pages of your Journal by these long extracts, because I thought, from the celebrity of the author, his work might be supposed to convey to us a pretty accurate summary of the practice on this particular point, among our continental brethren.

The extracts scarcely need a comment;—force, almost unlimited force, is the decided practice of Boyer. The power of "six," even "nine or ten!" men, is employed by him to do that, which he acknowledges has yielded to *less force in more unskilful hands!* And so far is the principle which I have endeavoured to establish, from governing the general practice of the French surgeons, that it is only resorted to by them, as a sort of dernier resource, "which has sometimes succeeded *after* all their *more violent* endeavours have failed them!

